# Severe West Nile Virus and Severe Acute Respiratory Syndrome Coronavirus 2 Infections in a Patient With Thymoma and Anti–Type I Interferon Antibodies

**DOI:** 10.1093/infdis/jiae321

**Published:** 2024-07-08

**Authors:** Federica Barzaghi, Camilla Visconti, Giovanni Battista Pipitone, Simone Bondesan, Giulia Molli, Stefania Giannelli, Claudia Sartirana, Vito Lampasona, Elena Bazzigaluppi, Cristina Brigatti, Adrian Gervais, Paul Bastard, Chiara Tassan Din, Chiara Molinari, Lorenzo Piemonti, Jean-Laurent Casanova, Paola Carrera, Giorgio Casari, Alessandro Aiuti

**Affiliations:** Pediatric Immunohematology and Bone Marrow Transplantation Unit, IRCCS San Raffaele Scientific Institute, Milan, Italy; San Raffaele Telethon Institute for Gene Therapy, IRCCS San Raffaele Scientific Institute, Milan, Italy; Pediatric Immunohematology and Bone Marrow Transplantation Unit, IRCCS San Raffaele Scientific Institute, Milan, Italy; San Raffaele Telethon Institute for Gene Therapy, IRCCS San Raffaele Scientific Institute, Milan, Italy; Vita-Salute San Raffaele University, Milan, Italy; Laboratory of Clinical Genomics, IRCCS San Raffaele Scientific Institute, Milan, Italy; Laboratory of Clinical Genomics, IRCCS San Raffaele Scientific Institute, Milan, Italy; Laboratory of Clinical Genomics, IRCCS San Raffaele Scientific Institute, Milan, Italy; San Raffaele Telethon Institute for Gene Therapy, IRCCS San Raffaele Scientific Institute, Milan, Italy; San Raffaele Telethon Institute for Gene Therapy, IRCCS San Raffaele Scientific Institute, Milan, Italy; San Raffaele Diabetes Research Institute, IRCCS San Raffaele Scientific Institute, Milan, Italy; San Raffaele Diabetes Research Institute, IRCCS San Raffaele Scientific Institute, Milan, Italy; San Raffaele Diabetes Research Institute, IRCCS San Raffaele Scientific Institute, Milan, Italy; Laboratory of Human Genetics of Infectious Diseases, Necker Branch, INSERM U1163, Necker Hospital for Sick Children, Paris, France; Paris Cité University, Imagine Institute, Paris, France; Laboratory of Human Genetics of Infectious Diseases, Necker Branch, INSERM U1163, Necker Hospital for Sick Children, Paris, France; Paris Cité University, Imagine Institute, Paris, France; St Giles Laboratory of Human Genetics of Infectious Diseases, Rockefeller Branch, The Rockefeller University, New York, USA; Pediatric Hematology-Immunology and Rheumatology Unit, Necker Hospital for Sick Children, Assistance Publique-Hôpitaux de Paris, Paris, France; Clinic of Infectious Diseases, Division of Immunology, Transplantation and Infectious Diseases, IRCCS San Raffaele Scientific Institute, Milan, Italy; San Raffaele Diabetes Research Institute, IRCCS San Raffaele Scientific Institute, Milan, Italy; Vita-Salute San Raffaele University, Milan, Italy; San Raffaele Diabetes Research Institute, IRCCS San Raffaele Scientific Institute, Milan, Italy; Laboratory of Human Genetics of Infectious Diseases, Necker Branch, INSERM U1163, Necker Hospital for Sick Children, Paris, France; Paris Cité University, Imagine Institute, Paris, France; St Giles Laboratory of Human Genetics of Infectious Diseases, Rockefeller Branch, The Rockefeller University, New York, USA; Pediatric Hematology-Immunology and Rheumatology Unit, Necker Hospital for Sick Children, Assistance Publique-Hôpitaux de Paris, Paris, France; Howard Hughes Medical Institute, Paris, France; Laboratory of Clinical Genomics, IRCCS San Raffaele Scientific Institute, Milan, Italy; Unit of Genomics for Human Disease Diagnosis, IRCCS San Raffaele Scientific Institute, Milan, Italy; Vita-Salute San Raffaele University, Milan, Italy; Genomic Unit for the Diagnosis of Human Pathologies, IRCCS San Raffaele Scientific Institute, Milan, Italy; Pediatric Immunohematology and Bone Marrow Transplantation Unit, IRCCS San Raffaele Scientific Institute, Milan, Italy; San Raffaele Telethon Institute for Gene Therapy, IRCCS San Raffaele Scientific Institute, Milan, Italy; Vita-Salute San Raffaele University, Milan, Italy

**Keywords:** COVID-19, West Nile virus, anti-IFN autoantibodies, thymoma, TLR3, CCR5

## Abstract

Patients with severe West Nile virus and SARS-CoV-2 infections deserve accurate diagnosis of underlying diseases, determining possible anti-interferon autoantibody production, since they must receive antiviral and immunological therapies to enhance antiviral response.

The current study aimed to investigate determinants of severity in a previously healthy patient who experienced 2 life-threatening infections, from West Nile Virus (WNV) and severe acute respiratory syndrome coronavirus 2 (SARS-CoV2). During coronavirus disease 2019 (COVID-19) hospitalization he was diagnosed with a thymoma, retrospectively identified as already present at the time of WNV infection. Heterozygosity for p.Pro554Ser in the *TLR3* gene, which increases susceptibility to severe COVID-19, and homozygosity for *CCR5* c.554_585del, associated with severe WNV infection, were found. Neutralizing anti-interferon (IFN)-α and anti-IFN-ω autoantibodies were detected, likely induced by the underlying thymoma and increasing susceptibility to both severe COVID-19 pneumonia and West Nile encephalitis.

The first line of defense against pathogens relies on innate immunity. Interferons (IFNs) play a key role in the initial antiviral immune response, binding to cell surface receptors and activating the expression of IFN-stimulated genes. Many inborn errors of immunity with an altered type I IFN-related response are associated with severe viral diseases [1].

Patients with severe coronavirus disease 2019 (COVID-19) have reduced IFN-mediated antiviral response, along with massive proinflammatory cytokine production [2]. Moreover, some germline loss-of-function variants in the type I IFN signaling pathway have been described in 2%–3% of severe life-threatening severe acute respiratory syndrome coronavirus 2 (SARS-CoV-2) infections [3]. Moreover, about 15% of life-threatening cases of COVID-19 infection have been related to neutralizing autoantibodies against IFN-ω and/or IFN-α. They can neutralize low (100 pg/mL), intermediate (1 ng/mL), or high (10 ng/mL) concentrations of type I IFNs, impairing their ability to block SARS-CoV-2 infection [4]. Autoantibodies neutralizing type I IFNs have been found in patients with systemic lupus erythematosus, in nearly all patients with autoimmune polyendocrinopathy-candidiasis-ectodermal dystrophy, and in almost 60% of patients with thymoma and myasthenia gravis [5]. Moreover, these autoantibodies are found in a minority of uninfected people aged >70 years (∼4%) but can also be found in younger people from the general population (0.18% between 18 and 69 years of age) [6]. Therefore, they should be investigated when an atypical infectious history is present even in patients without an underlying pathology.

West Nile virus (WNV) is an RNA virus causing a neuroinvasive disease in <1% of patients. Variants impairing the IFN signaling or chemokine receptors were hypothesized as possible risk factors [7], and a recent meta-analysis confirmed the role of CCR5Δ32 variant in severe WNV infection [8].

We describe a peculiar combination of genetic background and acquired autoantibody production predisposing to both severe West Nile and SARS-CoV-2 infections, suggesting crucial clinical implications in the identification and management of these patients.

## MATERIALS AND METHODS

The research immunological and genetic studies were performed in line with the principles of the Declaration of Helsinki.

### Genomic DNA Extraction From Peripheral Blood

Genomic DNA was extracted using Maxwell DNA blood kit (Promega) following the manufacturer's instructions on a Maxwell automatic platform.

### Genomic DNA Extraction From Formalin-Fixed, Paraffin-Embedded Thymoma Tissue

Genomic DNA extraction from formalin-fixed, paraffin-embedded (FFPE) tissue sections of thymoma after slide scraping of FFPE-selected tumoral areas (100%) was performed using Maxwell RSC DNA FFPE Kit (Promega) following the manufacturer's instructions.

### Quantification of Extracted DNA

DNA quantification were assessed using a Qubit Fluorometer with double-stranded DNA (dsDNA) HS Assay Kit for Qubit and dsDNA BR Assay Kit for Qubit (Thermo Fisher Scientific, Wilmington, Delaware).

### Next-Generation Sequencing

Whole exome sequencing was performed starting from 100 ng of genomic DNA. Library preparation was performed using SureSelect XT Low Input kit (Agilent). Target enrichment was performed through SureSelect Human All Exon V7 probes (Agilent). The libraries were sequenced on the NovaSeq 6000 platform, 2 × 150 bp (Illumina). Reads alignment and variant calling were performed by Dynamic Read Analysis for GENomics (DRAGEN) on BaseSpace Sequence Hub (Illumina) pipelines. BAM files were visualized with Integrative Genome Viewer software (Broad Institute). Variant annotation and prioritization were conducted using the eVai (EnGenome) tool allowing manually filtered variants, based on different sets of filters with variable stringency: high-quality (pass all quality filters) nonsynonymous variants, population frequency (minor allele frequency <2% or <1%), in silico gene panels, consequence/effect, and inheritance mechanism. The remaining variants were classified according to the American College of Medical Genetics (ACMG) standard, in silico predictions, and experimental data in the literature. Candidate variants with unknown significance (class 3), likely pathogenetic (class 4), or pathogenetic (class 5) were included in the study.

### Proliferative Response Assays

Peripheral blood mononuclear cells isolated by Ficoll gradient were cultured in X-VIVO 15 (Lonza) at 5% of human sera (EuroClone S.p.A.) alone or in the presence of polyclonal or antigenic stimuli. Tritiated thymidine was added to the medium 16–18 hours before the end of the culture. The proliferation of the activated lymphocytes was measured as count per minute by MicroBeta Counter (Trilux Perkin Elmer Life Sciences) and reported as stimulation index ratio between cells stimulated versus the cells cultured without stimuli. Evaluation of proliferative response to spike SARS-CoV-2 protein was performed with SARS-CoV-2 PepTivator peptide pools Prot_S (Miltenyi Biotec).

### SARS-CoV-2 Receptor-Binding Domain Antibody Luciferase Immunoprecipitation System Assay

IgG binding to the Wuhan SARS-CoV-2 spike protein receptor-binding domain (RBD) was measured by luciferase immunoprecipitation system using a NanoLuc (Promega) nanoluciferase-tagged recombinant antigen expressed in Expi293F (Thermo Fisher Scientific) eukaryotic cells. In brief, patient serum (1 μL) was incubated with nanoluciferase-RBD (2 million light units equivalents of luciferase activity) in Tris-buffered saline pH7.4 0.5% Tween (TBST) for 1 hour at room temperature in a 96-well plate. Immune complexes were then recovered by adding protein-A sepharose beads (Cytiva) and incubating with shaking for 1 hour at 4°C. To remove unbound antigen, sepharose beads were then washed 5 times by sequential centrifugation, TBST aspiration, and dispensing. After washing, the NanoLuc luciferase substrate (Promega) was added to the plate wells and light emission (single photon counting light units [LUs]) measured over a 2-second time span in a luminometer (Berthold GmbH). Raw data LUs were then converted into arbitrary units using a strongly immunized serum as reference. The assay threshold for positivity was placed at the 99th percentile of values measured in prepandemic control samples (n = 384).

### Luciferase Reporter Assay

The plasma blocking activity against type I IFNs (13 IFN-α subtypes, IFN-β, and IFN-ω) was determined with a luciferase reporter assay. HEK293T cells, cultured in Dulbecco’s modified Eagle medium (DMEM; Thermo Fisher Scientific) with 10% fetal bovine serum (FBS), were transfected in the presence of X-tremeGene9 transfection reagent (Sigma-Aldrich) for 24 hours with a human interferon-stimulated response element-luciferase plasmid in the pGL4.45 backbone and a plasmid constitutively expressing Renilla luciferase for normalization (pRL-SV40). Then, cells were left unstimulated or were stimulated with IFNs (IFN-α subtypes [PBL Assay Science], IFN-ω [Peprotech], or IFN-β [Peprotech]) at 1 ng/mL for 16 hours at 37°C in the presence of 10% of healthy control or patient plasma diluted in DMEM with 2% FBS. Luciferase activity was assessed in the Dual-Luciferase Reporter 1000 assay system. Raw luciferase induction was calculated as firefly luciferase activity normalized against Renilla luciferase activity, and this raw luciferase induction was normalized against the median induction of healthy controls without neutralizing autoantibodies.

## RESULTS

**Figure 1.**
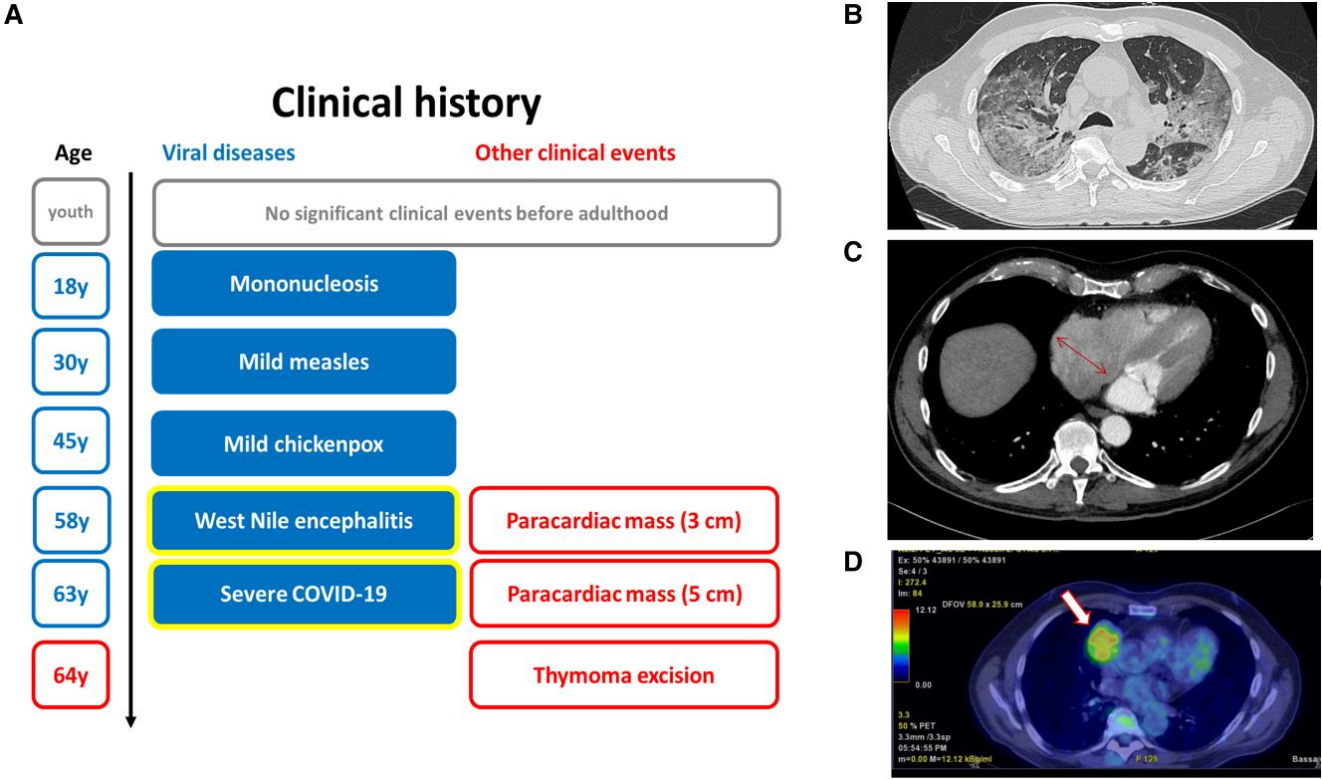
Patient's clinical history and imaging. *A*, Timeline of viral infections and identification of the mediastinal mass, then diagnosed as thymoma. *B*, High-resolution chest computed tomography (CT) scan showing coronavirus disease 2019 (COVID-19) pneumonia, characterized by bilateral ground-glass areas associated with interstitial thickening, most extensively in the upper lung lobes and areas of pulmonary consolidation with dense streaks in the lower lobes bilaterally. *C*, CT scan showing right paracardiac mass with maximum transverse diameter of 5 cm (arrow). *D*, Low-dose positron emission tomography/CT scan with [^18^F]fluorodeoxyglucose showing medium accumulation in the right paracardiac mediastinal mass (arrow).

A 63-year-old man admitted with severe COVID-19 infection, requiring mechanical ventilation, subsequently complicated with sepsis and pulmonary embolism. He slowly recovered after multiple nonspecific therapies (steroids, anakinra, antibiotics). His clinical history was characterized by infectious mononucleosis at 18 years of age, uncomplicated measles at 30 years of age, and chickenpox at 45 years of age (Figure 1*A*).

Remarkably, his past history revealed a life-threatening West Nile encephalitis at age 58 years. During this hospitalization, thorax CT scan identified a 3-cm paracardiac mass, which had increased up to 5 cm at the time of COVID-19 pneumonia (Figure 1*B–D*).

Considering the severe evolution of both infections (WNV and SARS-CoV-2) in the absence of other clear predisposing factors, despite age, genetic analysis was performed for the patient (I.1).

**Figure 2.**
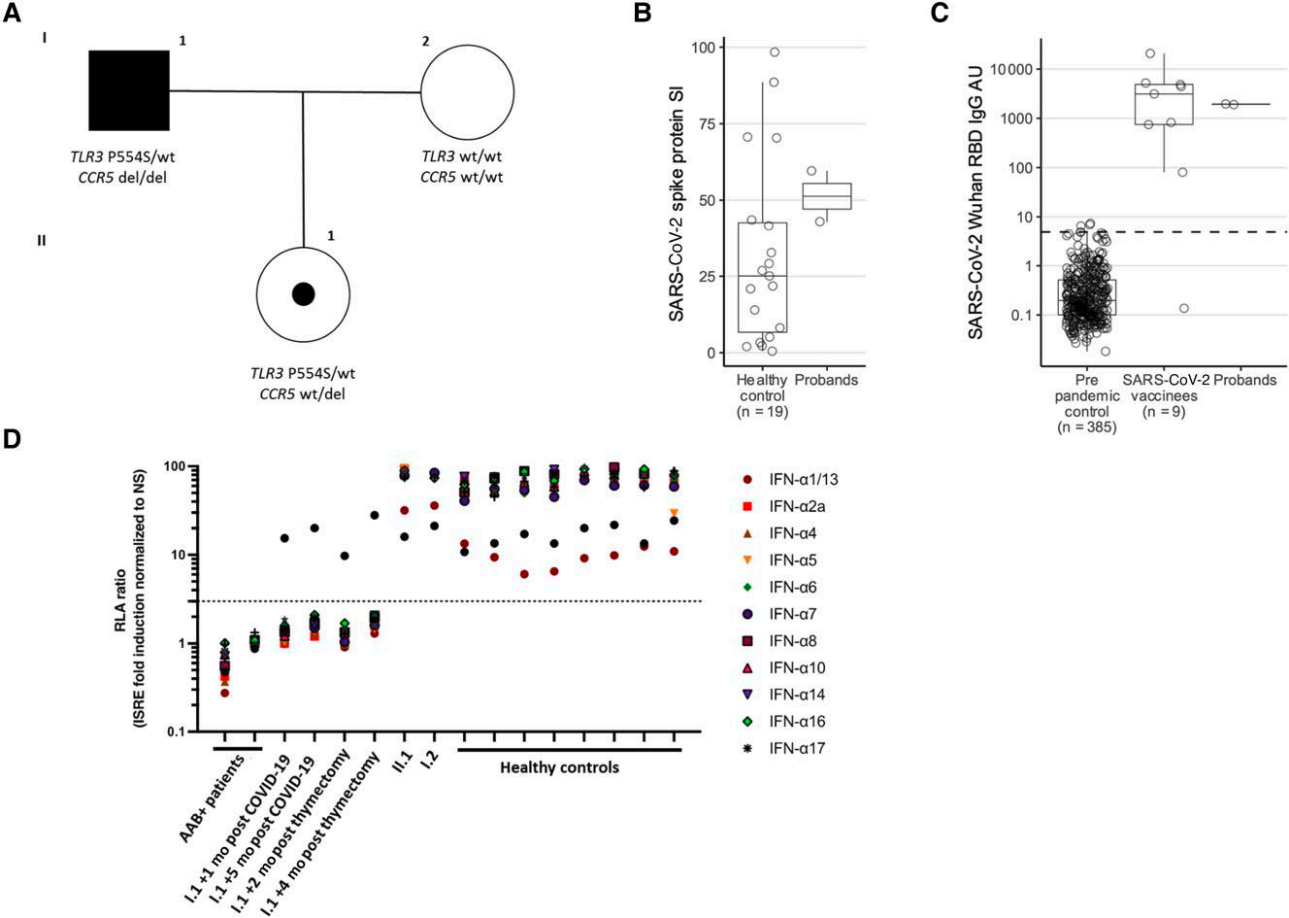
Genetics and immunological response to severe acute respiratory syndrome coronavirus 2 (SARS-CoV-2) of the patient and family. *A*, Pedigree and segregation of the *TLR3* variant c.2660C > T, p.P554S and of the *CCR5* variant c.554_585del, p.Ser185IlefsTer32 (CCR5Δ32). Proliferative response to SARS-CoV-2 spike protein peptides (*B*) and Wuhan spike receptor-binding domain immunoglobulin G levels (*C*) in the probands (I.1 and II.2) and 2 control cohorts; the assay cut-off is shown as a dashed line. *D*, Anti–type I interferon (IFN) autoantibodies: neutralization of type I IFNs (1 ng/mL of IFNs) detected in a luciferase-based cell reporter assay, in the patient plasma/serum (I.1) at different time points after coronavirus disease 2019 infection, before and after thymectomy, in the daughter (II.1) and in control groups (autoantibody-positive patients and healthy control without autoantibodies). Abbreviations: AAB, autoantibody; AU, arbitrary units; COVID-19, coronavirus disease 2019; IFN, interferon; IgG, immunoglobulin G; ISRE, interferon-stimulated response element; NS, not stimulated; RBD, receptor-binding domain; RLA, relative luciferase activity; SARS-CoV-2, severe acute respiratory syndrome coronavirus 2; SI, stimulation index; wt, wild type.

Whole exome sequencing (WES) analysis revealed the presence of 2 variants, both confirmed by Sanger sequencing—a heterozygous c.1660C > T rare variant on *TLR3* (NM_003265.2; rs121434431), with a 0.007 frequency in the European non-Finnish (gnomAD v2.1.1) and causing the missense change p.Pro554Ser (Figure 2*A*), classified as “likely pathogenic” according to ACMG. This specific *TLR3* variant has already been described in literature to be biochemically deleterious in the heterozygous state [3].

*TLR3* is a nucleic acid sensor that recognizes viral dsRNA and induces the activation of the innate immune response and the production of type I interferons. On the other hand, the patient also displayed the homozygous c.554_585del variant in *CCR5* (NM_000579.3; rs333) with a 1.09% frequency in the European non-Finnish population (gnomAD v2.1.1), causing the frameshift p.Ser185IlefsTer32 (Figure 2*A*). The ClinVar database defines the variant as benign because it is protective for human immunodeficiency virus (HIV) infection, but it is also reported as a risk factor affecting the clinical course of WNV infection [9], and the Leiden Open Variation Database classifies it as “pathogenic” because it affects the function of the gene.

Thus, the genetic study was extended to the patient's daughter (II.1) and wife (I.2), who experienced COVID-19 in a milder form. Only the daughter was found as a carrier for the *CCR5* deletion and the p.Pro554Ser variant on *TLR3* (Figure 2*A*).

Both I.1 and II.1 did not display major immunological abnormalities: Plasma immunoglobulins and specific antibody responses to previous infections/vaccinations, lymphocyte subsets on peripheral blood, and proliferative response in vitro to mitogens and antigens (alloantigens, *Candida*, tetanus toxoid, herpes simplex virus, cytomegalovirus) were normal. The patient (I.1) showed only mild reduction of total B-cell count (90 cells/μL [normal range, 110–570 cells/μL) and inversion of CD4:CD8 ratio (Supplementary Table 1). Moreover, both I.1 and II.1 developed adequate T-cell proliferative response in vitro (Figure 2*B*) and specific antibodies (anti-RBD) (Figure 2*C*) toward SARS-CoV-2.

Due to the absence of molecular etiology for severe COVID-19, we questioned whether other factors could induce such a severe clinical evolution. The answer came from the mediastinal mass, histologically diagnosed as type AB thymoma, which was metabolically active (Figure 1*D*).

The genomic profile of the tumor was analyzed through WES, and no candidate variants were identified. Considering the risk of autoantibody production related to thymoma, we demonstrated the presence of autoantibodies neutralizing 1 ng/mL IFN-α and anti-IFN-ω autoantibodies in the patient (not in the daughter), even 6 months after thymectomy (Figure 2*D*). Thus, these autoantibodies represented for the patient an additional risk factor for both severe COVID-19 and invasive WNV infections. Although they are more commonly found in older people, in this case the patient was respectively 58 and 63 years old when he suffered from these conditions, emphasizing the importance of the detection of these autoantibodies in this setting.

## DISCUSSION

The reported clinical case represents a unique example of combined genetic and acquired risk factors for severe COVID-19 and WNV infection. Indeed, our patient harbors the heterozygous (p.Pro554Ser) missense variant in the *TLR3* gene and is homozygous for the *CCR5Δ32* deletion, but he was also affected by a thymoma causing production of anticytokine antibodies.

TLR3 deficiency due to the p.Pro554Ser variant confers an autosomal dominant predisposition to herpes simplex virus encephalitis and to COVID-19 infection, with incomplete clinical penetrance [3]. The TLR3–IFN-α pathway is recognized to be relevant also for the response to WNV encephalitis. Indeed, NS1 protein of WNV inhibits TLR3 signal transduction to facilitate viral expansion [10]. TLR3 knockout mice have increased viral burden into the brain, compared to wild type [11]. Additionally, in humans, downregulation of TLR3 on macrophages in elderly persons seems to be related to an increased risk of impaired response to WNV [12].

The genetic analysis identified the *TLR3* p.Pro554Ser variant in the patient, who experienced severe WNV encephalitis and severe COVID-19, and in his daughter, who had a mild COVID-19 infection, supporting an incomplete clinical penetrance.

The patient was even homozygous for the CCR5Δ32 variant, which is known to confer resistance to HIV infection and is associated with severe course of WNV infection [8]. Similarly, *Ccr5*^–/–^ WNV-infected mice display impaired leukocyte trafficking toward the brain, reduced capacity of viral control, increased disease severity, and higher mortality rates than *Ccr5* wild-type mice [13]. These data support the role of CCR5 as a protective factor against WNV infection. However, CCR5Δ32’s contribution to the risk of contracting SARS-CoV-2 infection and its severe evolution remains still elusive in literature [14, 15]. Thus, lack of CCR5 expression may have different roles depending on the pathogen.

Our patient's genotype is a combination of factors without a certain predisposing effect toward the viruses causing the 2 life-threatening infections experienced (Supplementary Table 1).

A possible explanation arose from the mediastinal mass already identified at the time of WNV infection and later diagnosed as a thymoma. Indeed, thymoma can induce the production of several autoantibodies due to impaired AIRE expression and central tolerance within the lesion [16], and WNV infection has been reported in the setting of Good syndrome [17].

Despite the absence of other autoimmune manifestation, typical of thymoma (eg, myasthenia gravis), the patient produced anti-IFN-α and anti-IFN-ω autoantibodies, which contributed to the severe evolution of SARS-CoV-2 infection and might have favored invasive WNV infection. The odds ratio for WNV disease in individuals with these autoantibodies relative to those without them in the general population is much higher when both anti-IFN-α and anti-IFN-ω are present, even if at low concentration [18]. Moreover, the odds ratio for critical COVID-19 was similarly higher in the subset of patients with positivity for both autoantibodies [19].

This case suggests that autoantibodies neutralizing type I IFNs should be investigated in patients with thymoma and in individuals with severe viral diseases known to be associated with these autoantibodies (eg, varicella zoster virus disease [20], critical influenza pneumonia [21], Middle East respiratory syndrome [22]) in order to ensure an early diagnosis and targeted treatment favoring better outcomes. These autoantibodies represent acquired risk factors that, importantly, do not fade after thymectomy (Figure 2*D*). Hence, this should be considered a permanent vulnerability, despite competent adaptive immunity and full immunization against SARS-CoV-2. Interestingly, in a mouse model of WNV infection, administration of neutralizing antibody to anti-IFN receptor before viral challenge resulted in enhanced susceptibility to WNV [23]. Moreover, a recent large international cohort study showed that 40% of patients with WNV encephalitis carry autoantibodies neutralizing IFN-α and/or IFN-ω [18].

Overall, this is an example of how genetic and acquired factors may increase susceptibility and severity of specific infections, as similarly described for a patient affected by severe COVID-19 with both *TLR7* deficiency and anti-IFN autoantibodies [24]. Indeed, the association of the heterozygous missense *TLR3* variant, the homozygous *CCR5Δ32* deletion, and the production of anti-IFN type I antibodies likely contributed to the severe course of COVID-19 and WNV infection in this patient.

In conclusion, patients with such an immunological background deserve dedicated management due to their high risk of poor outcome related to a delayed clearance of the virus. This observation may pave the way to the application, in similar cases, of therapies with antivirals or IFNs not targeted by autoantibodies. Further cohort studies are needed to elucidate the effectiveness of these therapeutic approaches in anticipating the viral clearance.

## Supplementary Data

Supplementary materials are available at *The Journal of Infectious Diseases* online (http://jid.oxfordjournals.org/). Supplementary materials consist of data provided by the author that are published to benefit the reader. The posted materials are not copyedited. The contents of all supplementary data are the sole responsibility of the authors. Questions or messages regarding errors should be addressed to the author.

## Supplementary Material

jiae321_Supplementary_Data

## Notes

***Acknowledgments.*** We thank the patient and his family. We thank the Italian Primary Immunodeficiency network (IPINet) and the Project IEI-Net (PNRR-MR1-2022-12376594). The authors of this publication are members of the European Reference Network for Rare Immunodeficiency, Autoinflammatory and Autoimmune Diseases (ERN-RITA).

***Author contributions*.** F. B., C. V., and G. B. P. wrote the first draft of the manuscript. F. B., C. V., C. T. D., and C. M. clinically followed the patient and his family. F. B. and C. V. provided biological samples. G. B. P., S. B., and G. M. performed the genetic analysis. P. C. supervised the genetic analysis and guided the interpretation of the results. S. G., C. S., E. B., C. B., and A. G. performed the in vitro studies. A. G. and V. L. analyzed the results of in vitro studies. P. B. and J.-L. C. performed immunological studies and contributed to interpretation of the results. G. C. and A. A. supervised the work, contributed to interpretation of the results, and reviewed the manuscript. All authors critically revised and approved the final manuscript.

***Ethics approval.*** The research immunological and genetic studies were performed in line with the principles of the Declaration of Helsinki. Approval of the Protocol TIGET09, “Collection of biological material for the study of blood cells and their microenvironment and for the development of new therapeutic approaches to genetic diseases and cancers,” was granted by the San Raffaele Hospital Ethical Committee on 11 May 2017. The patient was enrolled in protocols COVID-BioB/Gene-COVID approved by the local ethical committee on 5 May 2020. Informed consent was obtained from all individual participants included in the study. The authors affirm that human research participants provided informed consent for publication of the images and clinical data herein contained.

***Data availability.*** The datasets generated during and/or analyzed during the current study are available in the National Institutes of Health (NIH) repository (Sequence Read Archive), accession number PRJNA964759 (https://www.ncbi.nlm.nih.gov/sra/?term=PRJNA964759).

***Financial support.*** This work was supported by Ministero della Salute (grant nr. COVID-2020-12371617) and Fondazione Telethon (core grant nr. TTAAB0222TT). The Laboratory of Human Genetics of Infectious Diseases is supported by the Howard Hughes Medical Institute, the Rockefeller University, the St Giles Foundation, the NIH (R01AI088364, R01AI127564, R01AI163029 and R21AI160576), the National Center for Advancing Translational Sciences, the NIH Clinical and Translational Science Award program (UL1TR001866), the Fisher Center for Alzheimer's Research Foundation, the Meyer Foundation, the JPB Foundation, the “Investissement d’Avenir” program launched by the French government and implemented by the Agence Nationale de la Recherche (ANR) (ANR-10-IAHU-01), the Integrative Biology of Emerging Infectious Diseases Laboratory of Excellence (ANR-10-LABX-62-IBEID), the French Foundation for Medical Research (FRM) (EQU201903007798), ANR GENVIR (ANR-20-CE93-003), ANR AI2D (ANR-22-CE15-0046) and ANR AAILC (ANR-21-LIBA-0002) projects, Horizon Europe UNDINE project (GA no. 101057100), the ANR-RHU COVIFERON Program (ANR-21-RHUS-08), the Square Foundation, Grandir–Fonds de solidarité pour l’enfance, the Fondation du Souffle, the SCOR Corporate Foundation for Science, the Battersea & Bowery Advisory Group, the French Ministry of Higher Education, Research, and Innovation (MESRI-COVID-19), Institut National de la Santé et de la Recherche Médicale (INSERM), REACTing-INSERM, and the University of Paris. P. B. was supported by the MD-PhD program of the Imagine Institute (with support from the Bettencourt-Schueller Foundation). P. B. was supported by a “Poste CCA-INSERM-Bettencourt” (with support from the Bettencourt-Schueller Foundation) and the FRM (EA20170638020). A. G. was supported by a French governmental grant from the French National Agency for Research as part of the “Investissement d’Avenir” program (ANR-10-LABX-62-01).
