# Supplementary material for: Severe West Nile Virus and Severe Acute Respiratory Syndrome Coronavirus 2 Infections in a Patient With Thymoma and Anti–Type I Interferon Antibodies
Source: J Infect Dis. 2024 Jul 8;231(1):e206–12. doi: 10.1093/infdis/jiae321 (PMC11793036; doi:10.1093/infdis/jiae321)
Supplement: jiae321_Supplementary_Data [file jiae321_supplementary_data.docx]

**Table S1. Possible influence of the genetic and acquired features of the patient on the evolution of WNV and SARS-CoV2 infections.**

|  | ***TLR3*** | ***CCR5*** | **ANTI-IFN Ab** |
| --- | --- | --- | --- |
| **SEVERE WNV INFECTION** | TLR3 possible risk factor based on preclinical data [[9](#_ENREF_9)] | CCR5Δ32 risk factor based on meta-analysis [[7](#_ENREF_7)] | possible risk factor based on preclinical and clinical data [[17](#_ENREF_17),[22](#_ENREF_22)] |
| **SEVERE COVID-19** | TLR3 Pro554Ser known predisposing factor with incomplete penetrance [[3](#_ENREF_3)] | debated risk factor [[13](#_ENREF_13),[14](#_ENREF_14)] | known risk factor [[4](#_ENREF_4)] |
